# Different Responses in Vascular Traits between Dutch Elm Hybrids with a Contrasting Tolerance to Dutch Elm Disease

**DOI:** 10.3390/jof8030215

**Published:** 2022-02-22

**Authors:** Michal Moravčík, Miroslava Mamoňová, Vladimír Račko, Ján Kováč, Miloň Dvořák, Jana Krajňáková, Jaroslav Ďurkovič

**Affiliations:** 1 Department of Phytology, Technical University in Zvolen, T.G. Masaryka 24, 96001 Zvolen, Slovakia; michal.moravcik@uksup.sk (M.M.); kovacj@tuzvo.sk (J.K.); 2 Department of Pesticide Registration, ÚKSÚP Bratislava, SNP 99, 96202 Vígľaš, Slovakia; 3 Department of Wood Science, Technical University in Zvolen, T.G. Masaryka 24, 96001 Zvolen, Slovakia; mamonova@tuzvo.sk (M.M.); racko@tuzvo.sk (V.R.); 4 Department of Forest Protection and Wildlife Management, Mendel University in Brno, Zemědělská 3, 61300 Brno, Czech Republic; milon.dvorak@seznam.cz; 5 Scion, 49 Sala Street, Rotorua 3010, New Zealand; jana.krajnakova@scionresearch.com

**Keywords:** bordered pits, earlywood vessel, *Ophiostoma novo-ulmi*, vascular architecture, X-ray micro-computed tomography

## Abstract

The ascomycetous fungus *Ophiostoma novo-ulmi* is the causative agent of the current Dutch elm disease (DED) pandemic, which has ravaged many tens of millions of European and North American elm trees. Host responses in vascular traits were studied in two Dutch elm hybrids, ‘Groeneveld’ and ‘Dodoens’, which show different vascular architecture in the secondary xylem and possess contrasting tolerances to DED. ‘Groeneveld’ trees, sensitive to DED, possessed a high number of small earlywood vessels. However, these trees showed a poor response to DED infection for the earlywood vascular characteristics. Following infection, the proportion of least vessels with a vessel lumen area less than 2500 µm^2^ decreased from 65.4% down to 53.2%. A delayed response in the increasing density of vessels showing a reduced size in the latewood prevented neither the rapid fungal spread nor the massive colonisation of the secondary xylem tissues resulting in the death of the infected trees. ‘Dodoens’ trees, tolerant to DED, possessed a low number of large earlywood vessels and showed a prominent and fast response to DED infection. Vessel lumen areas of newly formed earlywood vessels were severely reduced together with the vessel size : number ratio. Following infection, the proportion of least vessels with a vessel lumen area less than 2500 µm^2^ increased from 75.6% up to 92.9%. A trend in the increasing density of vessels showing a reduced size was maintained not only in the latewood that was formed in the year of infection but also in the earlywood that was formed in the consecutive year. The occurrence of fungal hyphae in the earlywood vessels that were formed a year following the infection was severely restricted, as revealed by X-ray micro-computed tomography imaging. Possible reasons responsible for a contrasting survival of ‘Groeneveld’ and ‘Dodoens’ trees are discussed.

## 1. Introduction

Wild trees of the genus *Ulmus*, showing a low demand for soil and climatic conditions, resistant to mechanical damage, and frequently cultivated in urban areas, were heavily affected during the two waves of Dutch elm disease (DED). This wilt disease is caused by ascomycetous fungi of the genus *Ophiostoma*. Numerous attempts to control the disease focused not only on the reduction in elm bark beetle (vector) populations and the application of fungicides but also on the development of resistance to DED through breeding programs [1,2,3]. Since the outbreak of DED in the 1920s, many bred cultivars with varying degrees of resistance were planted in an attempt to mitigate the negative impacts of the pandemics and to restore the previous abundance of elm trees across both the European and North American continents [2,3]. However, despite being resistant to *O*. *ulmi* (Buism.) Nannf., some of these early cultivars such as ‘Belgica’, ‘Commelin’, ‘Vegeta’ and others were ravaged during the second DED wave caused by *O*. *novo-ulmi* Brasier [4]. At present, *O*. *ulmi* is almost extinct as a result of being outcompeted by *O*. *novo-ulmi* and their hybrids [5]. Relative setback in searching for resistant populations was partially minimised by the introduction of elm species from Asia. Hybrids containing the Asian elm species in their parentage were found to be more resilient to cope with *O*. *novo-ulmi*. In addition to DED tolerance, some of these newly released cultivars also possessed high ornamental value, exhibited fast growth and were tolerant to variable edaphic or climatic factors [6]. Many of the benefits previously conceded to elms still show both aesthetic and high-quality material values, which maintain the procedures of breeding and selection of new resistant hybrids in progress [7]. Besides the common breeding techniques, genetic engineering approaches were recently applied. However, these approaches were not very efficient due mostly to difficulties in the establishment of genetically modified trees in the natural environment. Common breeding thus remains the most consistent way to combat DED [8]. As a result of this effort, some resistant elm clones such as ‘Dehesa de Amaniel’, ‘Rebella’, ‘Sapporo Autumn Gold’ and others are now registered as suitable germplasm to renew the lost populations of *Ulmus* in forests and urban areas [4,9,10,11]. However, there is still the risk of a new disease outbreak caused by the ongoing adaptations of fungi to new environmental factors. Areas of distribution for the two *O*. *novo-ulmi* subspecies, *novo-ulmi* and *americana*, overlap in certain places and crossing between these subspecies frequently occurs [12]. Currently, hybridisation or introgression always creates new locally adapted fungal hybrids competing with parental subspecies and possibly overcoming them [5].

*Ophiostoma novo-ulmi* is a vascular pathogen responsible for the wilting of leaves, branches and eventually the death of the infected trees, all caused by the loss of water conductivity in the vessels [13]. The pathogen surviving in the nutritionally poor environment of the vessel produces a fungal biomass blocking water flow, the toxins causing wilting, and the cellulolytic enzymes degrading the cell walls. Moreover, tyloses and gums are formed as a defence response of the infected tree. Taken together, the physical blockage and enzymatic degradation of conduit cell walls accompanied by air bubble formation (embolism) result in a limited water supply [14].

Tolerance to DED is a combined effect of constitutive traits (vascular anatomy and cell wall chemical composition) together with induced traits (antifungal compounds and occlusive compounds) [8]. If these traits are combined with the selection of an early dormancy release and an early period of bud flushing, which do not overlap with the arrival of the pathogen vector, the infected tree contains enough metabolites to tolerate a fungal attack and to survive [15,16]. Such a tree might also benefit from the earlier formation of smaller vessels in the earlywood. The vascular characteristics, e.g., the size of vessels, have long been identified as a key factor affecting the resistance of elm trees to fungal infection [8]. Less vulnerable narrow vessels are less prone to cavitation and keep the water stream continuous for a longer period during the imposed stress. Narrow vessels are greater in number and easily exchangeable by neighboring vessels. If tyloses are also formed, this process occurs even faster and more effectively compared to that in the wider, more vulnerable vessels [17,18]. Vessel lumen area and vessel lumen fraction determine both the stem-stored water use and the pathogen spread [19]. The rate of pathogen spread is also regulated by pit membrane pore size and pit density, as larger membrane pores may enhance and smaller ones limit hyphae growth [20]. Another component of tolerance appears to be connected with metabolism as tolerant elms show a higher photosynthetic rate and have larger leaves with greater hydraulic conductivity and gas exchange [21]. Lignin monomer composition affects the extent of polysaccharide degradation by fungal cellulolytic enzymes in the cell walls of vascular conduits, and thus through the denser steric protection of readily degradable amorphous regions of polysaccharides, it may restrict the radial spread of the fungus [22]. An important but less frequently studied component of tolerance is the production of various chemical compounds possessing antifungal, antimicrobial and deterrent activities, including terpenoids and phenolics [23].

The objective of this study was to assess host responses to DED in vascular characteristics between ‘Groeneveld’ trees (sensitive to DED) and ‘Dodoens’ trees (tolerant to DED). We investigated the changes in vessel size and density that resulted from host responses in cambial activity following the artificial inoculation with the hybrid isolate of *O*. *novo-ulmi*.

## 2. Materials and Methods

### 2.1. Plant Material, Fungal Isolate and Study Site

Two Dutch elm hybrid cultivars with contrasting responses to DED were used, i.e., sensitive trees of ‘Groeneveld’ [(*Ulmus* × *hollandica* 49) × *U*. *minor* ssp. *minor* 1] [24] and tolerant trees of ‘Dodoens’ (open-pollinated *U*. *glabra* ‘Exoniensis’ × *U*. *wallichiana* P39) [25]. Ten-year-old micropropagated trees were artificially inoculated with a hybrid isolate of *O*. *novo-ulmi* ssp. *americana* × *novo-ulmi*, mating type B (deposited in the Culture Collection of the Department of Forest Protection and Wildlife Management, Faculty of Forestry and Wood Technology, Mendel University in Brno, Czech Republic, strain identifier: MENDELU1746). In order to induce the most authentic and contemporary infection conditions, the new strain of the pathogen was isolated from a dying *U*. *glabra* tree growing in a severely DED-infected locality at Brno-Komárov, Czech Republic (49°10′27″ N, 16°37′56″ E, 196 m a.s.l.) three weeks before the inoculations. Debarked pieces of the infected wood were sterilised with a solution of 7% sodium hypochlorite and 96% ethanol and subsequently were placed on a 3% malt extract agar. After two weeks of fungal growth, a portion of the mycelium was subjected to DNA extraction and then to fungal subspecies determination using a PCR-RFLP procedure [26] together with the mating type test determination proceeded by the cultivation method [27]. Another portion of the fungal mycelium was transferred into Tchernoff’s liquid medium (see details in Solla et al. [28]), and after three days of agitation, a yeast-like phase shake liquid culture was obtained with the concentration of 1.6 × 10^7^ spores per ml. The culture was diluted with distilled water to prepare an inoculation suspension of 10^6^ spores per ml. After one and a half days, the suspension (two drops per tree) was inoculated into a wound made by a scalpel in the current annual growth ring, 20 cm above the base of the stem. The inoculations were performed circa 30 days following the full leaf size development according to the instructions of Solla et al. [28]. Both inoculated and unwounded control trees were growing in an experimental field plot at Banská Belá, Štiavnické vrchy Mts., Slovakia (48°28′ N, 18°57′ E, 590 m a.s.l.) that represent a typical habitat for the scattered native *U*. *glabra* and *U*. *minor* taxa but free of any natural DED-infection source in the vicinity. The climate of the area is characterised by a mean annual temperature of 7.7 °C and a mean annual precipitation rate of 831 mm [21].

### 2.2. Wood Sampling

For ‘Groeneveld’ trees, the sampling was carried out six months following the inoculation because the infected trees unexpectedly died shortly after the completion of the current annual growth ring. For ‘Dodoens’ trees, growing vigorously to the present time and showing no signs of physiological, vascular or nanomechanical weakening several years following the inoculation [21,29], the sampling was carried out 42 months following the inoculation. In this case, we wanted to assess the vascular responses in the consecutive annual growth ring following inoculation to reveal how tolerant trees cope with the DED infection during the later period following inoculation. Four-centimeter-thick discs were sawn from the trunks at the height of 1 m above the base of the stem. The discs were symmetrical and did not contain knotwood or reaction wood. At the height of 1 m, the DED infection zones with typical dark staining of the wood were concentrated within the sixth annual growth ring (i.e., the outermost ring) in both Dutch elm hybrids. Vascular characteristics were determined for both the non-infected control wood and the infected wood zones of the sixth annual growth ring (in both hybrids), as well as for both the non-infected control wood and the newly formed wood of the seventh annual growth ring one year following the infection (in ‘Dodoens’ trees only). The experiments were conducted on the above wood samples taken from three infected and three non-infected trees per hybrid.

### 2.3. Scanning Electron and Fluorescence Microscopies

Wood sections (transverse, radial and tangential surfaces) were cut using the sledge microtome (Reichert, Vienna, Austria), mounted on specimen stubs, sputter-coated with gold and examined by high-vacuum scanning electron microscopy (SEM) using a VEGA TS 5130 instrument (Tescan, Brno, Czech Republic) operating at 15 kV.

For fluorescence microscopy observations, twenty-micrometer-thick cross-sections were mounted on glass slides in a drop of sterile water and examined using a Leica DM4000 B epifluorescence microscope (Leica Microsystems, Wetzlar, Germany). Autofluorescence of woody tissues was detected by excitation at 450 nm and emission at 515 nm using a Leica H3 filter cube (Leica Microsystems).

### 2.4. Determination of Vascular Traits

Vascular characteristics were determined from SEM images for the sixth annual growth ring (both hybrids) and the seventh annual growth rings (‘Dodoens’ trees only) using the NIS-Elements AR 3.0 image analysis software (Laboratory Imaging, Prague, Czech Republic) as described by Ďurkovič and Mišalová [30]. The traits such as vessel lumen area (*A*), vessel density per square millimeter of wood (*N*), radial and tangential diameters of vessels were determined for earlywood and latewood separately. Radial and tangential sizes were measured on 70 and 150 randomly selected vessels of earlywood and latewood, respectively. Additional vascular characteristics such as vessel lumen fraction (*F* = *A* × *N*) and the vessel size : number ratio (*S* = *A*/*N*) were also inferred for both earlywood and latewood as described in Zanne et al. [31]. Radial wall surfaces of earlywood vessels (at least 60 µm in diameter) were used for the determination of intervessel bordered pit aperture characteristics such as aperture area, maximum and minimum diameter of the aperture, the shape of aperture quantified as the circularity ratio and the abundance of bordered pits per 1000 µm^2^ of the vessel wall.

### 2.5. X-ray Micro-Computed Tomography

X-ray micro-computed tomography imaging of earlywood vessels was performed with a Phoenix V|Tome|X L 240 device (GE Sensing and Inspection Technologies, Wunstorf, Germany) as described in detail by Karadžić et al. [32]. Three-dimensional data sets were evaluated using VGSTUDIO MAX 2.2 software for industrial computed tomography data (Volume Graphics, Heidelberg, Germany).

### 2.6. Statistical Analysis

Data were analysed by one-way analysis of variance using SAS/STAT 9.1 software (SAS Institute, Cary, NC, USA). Duncan’s multiple range test was used for pairwise comparisons of means. However, the distribution of vessel lumen areas was left-skewed; therefore, the differences in this trait between non-infected and infected trees were analysed by the nonparametric Kolmogorov–Smirnov test. The results were considered statistically significant at α = 0.05.

## 3. Results

### 3.1. Earlywood Vascular Characteristics

**Figure 1:**
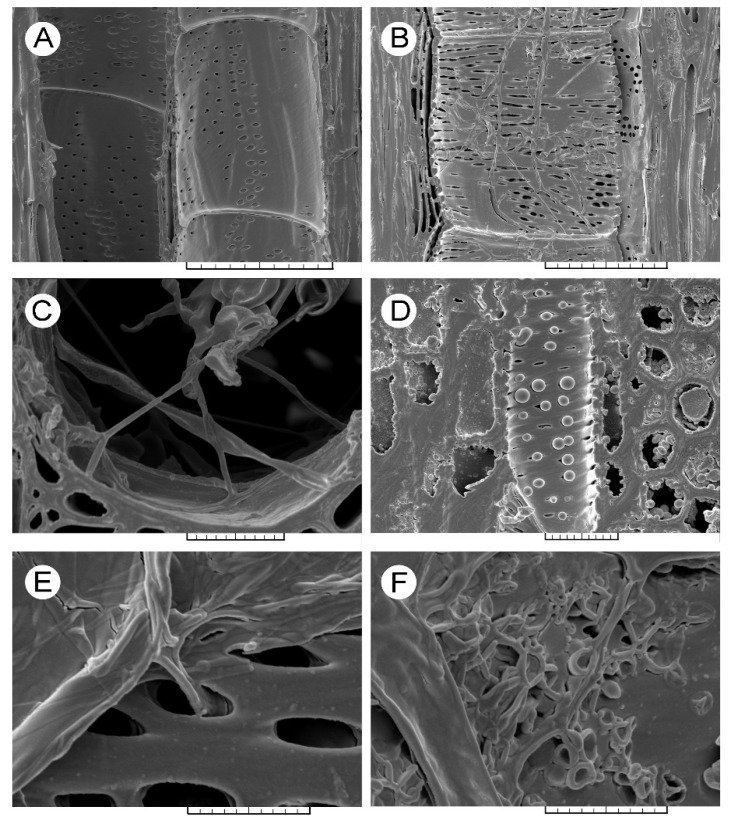
Scanning electron microscopy images of earlywood vessels in the sixth annual growth ring of ‘Groeneveld’ trees. (**A**) The non-infected tree, radial section, scale bar = 100 μm. (**B**) Densely spreading hyphae on the radial wall surface of an earlywood vessel, radial section, scale bar = 100 μm. (**C**) Both minute and large fungal hyphae spread through the lumen of an earlywood vessel, cross-section, scale bar = 20 μm. (**D**) A large number of small tyloses formed on the tangential wall surface of an earlywood vessel that was not capable of efficiently plugging the conduit, tangential section, scale bar = 20 μm. (**E**) The free penetration of the fungal hypha in an earlywood vessel, unrestricted by the bordered pit aperture size, radial section, scale bar = 10 μm. (**F**) Vegetative sporulation of the fungus on the radial wall surface of an earlywood vessel, radial section, scale bar = 10 μm.

**Figure 2:**
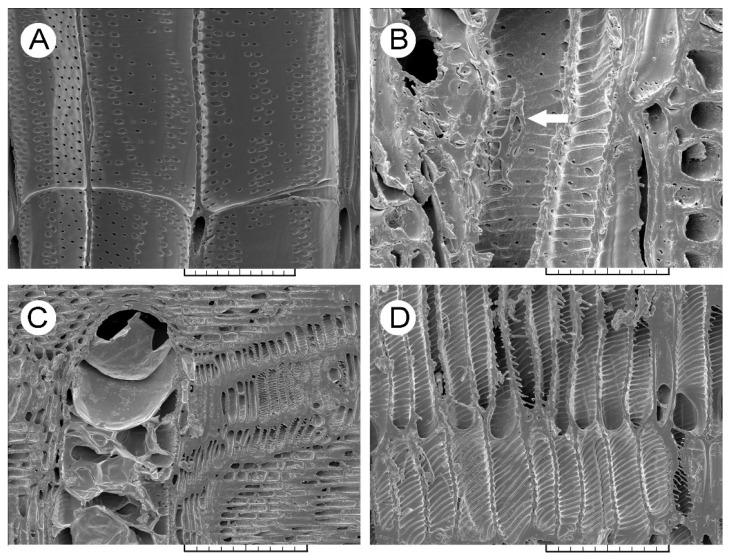
Scanning electron microscopy images of earlywood vessels in the sixth annual growth ring of ‘Dodoens’ trees. (**A**) The non-infected tree, radial section, scale bar = 100 μm. (**B**) The hyphae (white arrow) spread on the radial wall surface of an earlywood vessel, radial section, scale bar = 50 μm. (**C**) An earlywood vessel occlusion through tyloses following the fungal infection, radial section, scale bar = 200 μm. (**D**) Rapid formation of a large number of narrow earlywood vessels as the immediate response to the fungal infection, radial section, scale bar = 100 μm.

In the sixth annual growth ring of the non-infected trees (Figure 1A and Figure 2A), ‘Groeneveld’ showed a smaller earlywood vessel lumen area than ‘Dodoens’ (Table 1). Significantly smaller radial and tangential vessel diameters, as well as a lower *S* ratio and a greater number of vessels per unit area, were found for the non-infected trees of ‘Groeneveld’. The most abundant proportion of vessels was found in the size class of a vessel lumen area less than 2500 µm^2^ (65.4% for ‘Groeneveld’ and 75.6% for ‘Dodoens’, respectively; Figure S1A,B). The proportion of larger earlywood vessels (over 15,000 µm^2^) was higher for ‘Dodoens’ than for ‘Groeneveld’ trees. The most obvious difference in the proportion of vessels in individual vessel lumen area classes was observed for the size class over 27,500 µm^2^ that was fully missing in ‘Groeneveld’ trees, whereas this class was found to be the second most abundant type in ‘Dodoens’ trees (5.9%).

Upon infection, ‘Groeneveld’ trees (Figure 1B–F) responded with a decrease in the vessel lumen area of newly formed vessels in the earlywood of the sixth annual growth ring. However, this decrease in lumen area size was not so severe nor as prominent a response to the infection as that found for the infected ‘Dodoens’ trees (Table 1). Furthermore, the number of vessels per unit area, *S* ratio, as well as both the radial and tangential vessel diameters were not significantly changed in the infected trees of ‘Groeneveld’. The proportion of vessels with a vessel lumen area less than 2500 µm^2^ decreased from 65.4% down to 53.2%, whereas, in the size class of 2500–5000 µm^2^, the proportion increased from 5.9% up to 19.4% (Figure S1A). The overall fungal invasion of the sixth annual growth ring in the infected trees of ‘Groeneveld’ may be clearly seen in Video S1. On the contrary, in the earlywood of the sixth annual growth ring in the infected trees of ‘Dodoens’ (Figure 2B–D), vessel lumen areas of newly formed vessels were severely reduced (Table 1). Significant decreases were also found for both the radial and tangential vessel diameters, and the *S* ratio that was calculated at the 2.8-times increased in the number of vessels per unit area. The proportion of vessels in the size class of a vessel lumen area less than 2500 µm^2^ increased from 75.6% up to 92.9% (Figure S1B). The proportion of vessels in the size class of 2500–5000 µm^2^ was the second most abundant and increased from 1.0% up to 2.1%. For all the remaining vessel lumen area classes, the proportion of vessels was always lower than 1.2%, and the size class comprising the largest vessels (over 27,500 µm^2^) disappeared completely. The overall fungal invasion of the sixth annual growth ring in the infected trees of ‘Dodoens’ may be clearly seen in Video S2. In addition, the vessels and the surrounding parenchyma cells were frequently filled with inorganic crystals. The elemental composition analysis revealed that the crystals were composed mostly of calcium and silicon (Figure S2). Interestingly, in the sixth annual growth ring of the infected trees of ‘Dodoens’, distinct depositions of autofluorescent compounds were also observed in the parenchyma cells adjacent to the invaded vessels of earlywood (Figure S3B). However, in the infected trees of ‘Groeneveld’, the deposition of these autofluorescent compounds was seen at low intensity (Figure S3A).

Since the ‘Groeneveld’ trees did not survive the infection and died following the completion of the sixth annual growth ring, the vascular trait data of the seventh annual growth ring were measured on ‘Dodoens’ trees only. A tendency in vascular responses set in the sixth annual growth ring was also maintained in the seventh annual growth ring. The infected trees produced the highest observed number of earlywood vessels per unit area. Furthermore, vessel lumen area, radial and tangential diameters and the *S* ratio were also significantly decreased (Table 1). The proportion of vessels in the vessel lumen area class less than 2500 µm^2^ was again the most abundant (96.2%), whereas the size classes over 22,500 µm^2^ were missing (Figure S1C). The restricted occurrence of fungal hyphae in the seventh annual growth ring of the infected trees of ‘Dodoens’ may be clearly seen in Video S3.

In the non-infected trees, ‘Groeneveld’ earlywood vessels contained intervessel bordered pits that showed significantly smaller values than those of ‘Dodoens’ for the traits such as aperture area, the minimum diameter of the aperture, circularity ratio (i.e., the shape of these pits was more oval) and abundance per unit area of the cell wall (Table 2). Upon infection, ‘Groeneveld’ trees showed an increased circularity ratio (i.e., the shape of pits was less oval) and a higher abundance of pits per unit area. However, the infected ‘Dodoens’ trees showed significantly decreased values for the aperture area and minimum diameter of the aperture. In addition, these trees also changed the shape of pits to be less roundish, whereas the abundance of pits did not change. In the seventh annual growth ring of the infected ‘Dodoens’ trees, the aperture area and both the maximum and minimum diameters of the apertures were decreased. Interestingly, the abundance of pits achieved the highest observed value.

### 3.2. Latewood Vascular Characteristics

**Figure 3:**
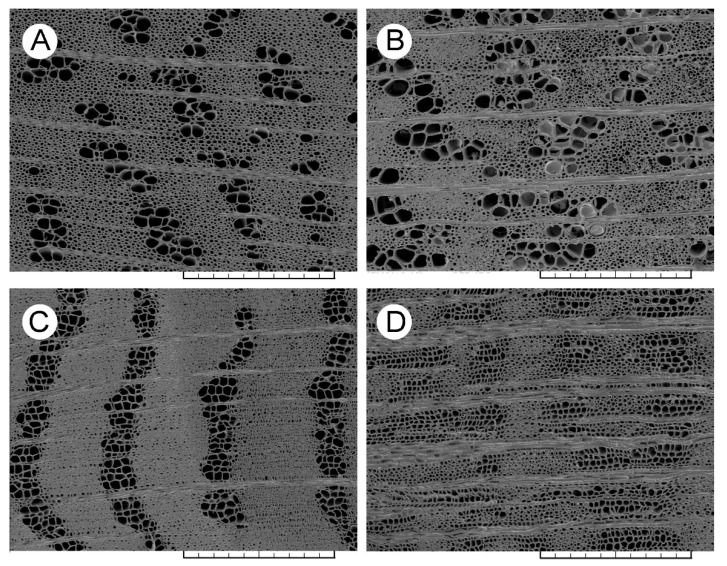
Scanning electron microscopy images of the latewood vascular architecture in the sixth annual growth ring of the examined Dutch elm hybrids. (**A**) The non-infected tree of ‘Groeneveld’. (**B**) The infected tree of ‘Groeneveld’. (**C**) The non-infected tree of ‘Dodoens’. (**D**) The infected tree of ‘Dodoens’. Cross-sections, scale bars (**A**–**D**) = 500 μm.

In the latewood of the sixth annual growth ring, the infected trees of ‘Groeneveld’ showed significant decreases in both vessel lumen area and in the *S* ratio. Contrary to the earlywood, the number of latewood vessels per unit area was significantly increased (Table 3). Moreover, the proportions of individual vessel lumen area classes were changed as a response to the infection (Figure S4A). In the size classes comprising the least vessels (lumen areas up to 600 µm^2^), the proportions of vessels substantially increased. In the size classes from 600 to 900 µm^2^, the proportions ranged from 8.0% to 9.9%. The size classes over 900 µm^2^ were less abundant, and their proportions were lower than 15.0% in total. Overall, the infection resulted in reducing the amount of larger latewood vessels, whereby the most abundant classes were those of the size less than 450 µm^2^ (54.0% in total). In the latewood of the sixth annual growth ring of the infected trees of ‘Dodoens’, vessel lumen area was again severely reduced following significant decreases in both the radial and tangential vessel diameters (Table 3). The number of vessels per unit area was substantially increased. The size classes less than 300 µm^2^ were the most abundant (87.0% in total; Figure S4B). The proportions of vessels with a size larger than 900 µm^2^ were negligible (0.6% in total). Scanning electron microscopy images of the latewood for both Dutch elm hybrids are presented in Figure 3A–D, including the control trees and the infected trees showing a modified vascular architecture in the latewood, especially for ‘Dodoens’ trees.

In the latewood of the seventh annual growth ring, the infected trees of ‘Dodoens’ showed a decreased number of vessels compared to the non-infected trees (Table 3). Although both the vessel lumen area and the vessel diameters were still significantly smaller compared to the non-infected trees, the mean vessel lumen area remained higher than that found for the sixth annual growth ring of the infected trees. These responses indicate the formation of an intermediate vascular architecture in the latewood tending to achieve vascular anatomy untouched by the infection. With regard to the proportion of vessels in each individual vessel lumen area class, the size class of 150–300 µm^2^ was the most abundant (Figure S4C).

## 4. Discussion

Vascular characteristics may influence the penetration, spread and overall negative impact of *O*. *novo-ulmi* isolates in the infected trees of *Ulmus*. From the two examined Dutch elm hybrids, ‘Dodoens’ trees survived the DED infection and continue to grow vigorously to the present day, contrary to the ‘Groeneveld’ trees that died several months following the inoculation. Previously reported differences in the physiological responses to DED between the two hybrids may explain their contrasting behaviour, including the changes and differences in leaf vascular, ecophysiological and leaf midrib nanomechanical traits, as well as the cell wall chemical composition of the secondary xylem [33]. However, constitutive traits such as vessel size and number are also involved in plant defence responses [34]. Some of these vascular characteristics are initially set before the onset of infection, whereas the modified properties of newly differentiated cells after the infection may be included in host responses to the pathogen. The ability to withstand a limited water supply, caused by the reduction in xylem transport capacity following the infection, functions as one of the key mechanisms for survival. Ďurkovič et al. [21] reported that ‘Dodoens’ trees had a significantly higher relative hydraulic conductivity compared to ‘Groeneveld’ trees that showed higher values for instantaneous water-use efficiency. That result may be supported by microscopical observations of larger earlywood vessels in the non-infected trees of ‘Dodoens’ compared to ‘Groeneveld’. Previous studies suggest a stronger effect and a more negative impact produced by *O*. *novo-ulmi* on trees with wide vessels, whereas trees possessing narrow vessels were less severely affected. Pita et al. [19] studied four *U*. *minor* clones with varying susceptibility to *O*. *novo-ulmi* and found that susceptible clones showed a higher theoretical hydraulic conductance than resistant ones. Different *U*. *minor* clones with variable susceptibility were more resistant when possessing vessels with a smaller diameter than clones with wider vessels [17]. The most susceptible *U*. *minor* trees possessed the highest proportion of vessels with a mean diameter larger than 100 µm [35]. Similarly, resistant *U*. *pumila* and *U*. *minor* trees showed vessels smaller in diameter compared to susceptible *U*. *minor* trees [20]. The relationship between tolerance/susceptibility to DED and the size of vascular conduits was also reported for other members of the Ulmaceae family (such as *U*. *glabra*, *Zelkova carpinifolia* and *Celtis australis*), among which the most tolerant *Celtis australis* showed the smallest vessel diameters [36]. It appears that the differences in tolerance among various elm trees are caused by the fact that the fungus and toxins can spread easily through wide vessels and, simultaneously, narrow vessels are more effectively and faster blocked by tyloses or other materials which prevent the spread of the fungus. In narrow vessels, the formation of these blocking structures has only a limited impact on the overall hydraulic conductivity [7].

Although previously published results indicate that trees possessing vessels with a smaller diameter obviously show a higher tolerance to DED [37,38], the circumstances are far more complicated. Following the crosses between resistant and sensitive maternal plants of *U*. *minor*, Martín et al. [37] found varying vessel sizes in the offspring with a different degree of tolerance to DED. All sensitive trees showed wide earlywood vessels, but not all tolerant trees had only narrow earlywood vessels. Venturas et al. [38] reported a relationship between the susceptibility to *Ophiostoma* infection and the vessel diameter in *U*. *minor*; however, a relationship between the resistance to cavitation and the tolerance to DED missed any significant correlation.

The earlywood vascular traits are much more critical for DED resistance than latewood traits because the time of earlywood formation correlates highly with an elm bark beetle attack and possible natural infection. During the period of maximum beetle attack, the trees of *U*. *minor* with large vessels were more sensitive to DED infection than *U*. *pumila* trees, which form narrower and denser vessels at the same time [39]. In light of the previously mentioned studies, our results of the earlywood vessel characteristics untouched by the infection would indicate the ‘Groeneveld’ trees to be a potentially tolerant clone possessing a high number of small vessels in the earlywood. However, this assumption has not been confirmed. ‘Dodoens’ trees, possessing larger earlywood vessels, coped quite effectively with DED and much better than ‘Groeneveld’ trees with smaller vessels.

In other pathosystems investigating tracheomycoses, there is an overall tendency to damage hosts with wide vessels more severely than hosts possessing narrow vessels. The infection and spread of *Phaeomoniella chlamydospora* in various cultivars of *Vitis vinifera* was more damaging to cultivars with a higher proportion of wider vessels than cultivars possessing narrower vessels. Moreover, the formation of tyloses and compartmentalisation of lumen space was less effective in wide vessels than in narrow ones [40]. *Ceratocystis lukuohia*-induced wilting of *Metrosideros polymorpha* trees is another example. This tropical tree does not form typical annual growth rings, but the outer sapwood vessels are naturally wider and contribute significantly to the susceptibility of a tree [41]. In addition, *Bretziella fagacearum*, a fungus infecting oak trees and causing oak wilt, differently affects a group of red oaks and a group of white oaks. The former is represented by highly susceptible species that die within a few weeks following infection. The latter are also susceptible species, their branches may wilt, but the trees often survive. Red oaks have rounded, thick-walled, and wider latewood vessels compared to the thin-walled, narrower vessels in white oaks [42]. The third group of oaks, evergreen or live oaks, shows an intermediate tolerance to wilt-inducing fungi. Despite sharing some characteristics typical of tolerant white oaks, the evergreen oaks resemble the red oaks more when considering their vessel anatomy. This similarity probably reflects a certain degree of susceptibility also present in the group of evergreen oaks [43]. Furthermore, oil palms (*Elaeis* spp.) infected by *Fusarium oxysporum* formed new stunted leaves after the onset of infection. Compared to the older leaves developed before the infection, these smaller leaves had narrow vessels to reduce the risk of embolism caused by the fungal toxins and enzymes [44]. Dutch elm hybrids examined in this study behaved this way only in response to the infection. In the case of the non-infected control trees, ‘Dodoens’ would seem to be less tolerant and, due to its large vessels, prone to a rapid spread of infection. However, upon infection, ‘Dodoens’ trees responded rapidly by changing the vessel lumen area and forming a greater number of vessels with narrow diameters in both the earlywood and latewood during the growing season in the year of the infection (i.e., the sixth annual growth ring). These changes were also found in the earlywood that was formed in the consecutive year (i.e., the seventh annual growth ring). The latewood of the seventh annual growth ring showed a moderate tendency to restore the vascular architecture to that before the infection.

As several previous studies suggest, the survival of infected trees appears to be connected with the size of the vessels [45,46,47]. An assessment of constitutive traits, such as vessel characteristics before or at the time of infection, is needed and should be supplemented with the study of induced responses in cambial activity and the production of new vessels that were differentiated under specific stimuli. Both of the tree species, avocado (*Persea americana*) and swamp bay (*Persea palustris*), are susceptible to laurel wilt disease caused by *Raffaelea lauricola*, and possess larger vessel lumen areas than the resistant camphor tree (*Cinnamomum camphora*) with smaller vessel lumen areas [45]. Similarly, avocado cultivars with different susceptibility to *R*. *lauricola* show different vessel diameters. The most sensitive cultivars have both larger vessel diameters and hydraulic conductivity than the less susceptible cultivars [46]. However, when comparing swamp bay, avocado and another closely related species, *Persea borbonia*, swamp bay is less sensitive to the pathogen despite possessing vessels with large diameters. Contrary to the genus *Ulmus*, species of the genus *Persea* have diffuse-porous wood forming same-sized vessels during the entire growing season. Although the vessels are larger and more prone to become severely invaded by fungi, the remaining vessels may substitute large ones to maintain optimal water transport capacity [47].

According to the mechanism of pathogen spread, not only are vessel size and diameter relevant to the progression of the disease, but bordered pit apertures in the cell wall also play some role. A low abundance of pits and the formation of pits with a smaller diameter may restrict the spread of the fungi. Resistant *U*. *pumila* and *U*. *minor* clones formed pits possessing both a smaller aperture diameter and aperture area when compared to the susceptible *U*. *minor* clones. Moreover, susceptible clones of *U*. *minor* showed a higher density of the pits in the vessel wall than that in the resistant clones [20]. As pits represent an important entrance point for hyphae penetration from one vessel to another, the size of the aperture may allow or limit (or at least decrease) the ability of the fungus to spread and colonise the woody tissues. In the xylem, the transport of fungal spores is a passive process, but when the spores reach the pit chambers, the hyphae begin to differentiate from the spore so that the fungal spread continues on through the pit membrane pores [48]. In the xylem of the host tree, the size of *Ophiostoma* spores may reach up to approximately 0.5 μm [49]; thus, the fungal spread may be limited by the size of the aperture. However, minor differences in bordered pit parameters found between the infected trees of the two Dutch elm hybrids can only barely explain their contrasting tolerance to DED. Przybył et al. [50] reported that *O*. *novo-ulmi* isolates produce cellulolytic enzymes that were later found to primarily degrade medium molecular weight macromolecules of cellulose in the cell walls of both Dutch elm hybrids [22]. This previous result supplemented with the current observations of frequent small tyloses inside vessels that were not capable of efficiently plugging the conduits could explain the unrestricted spread of fungal hyphae through the bordered pits, especially in the infected trees of ‘Groeneveld’. Moreover, the hyphae were also found in the lumens of vascular tracheids [22] and in ray parenchyma cells. In the case of the infected trees of ‘Dodoens’, we hypothesise that an enhanced abundance of autofluorescent defensive compounds such as tannins, lignans and suberins in the cells imminently surrounding the invaded vessel conduits might restrict radial fungal spread. Indeed, Ďurkovič et al. [22] reported substantially increased amounts of extractive compounds for the infected trees of ‘Dodoens’, whereas the infected trees of ‘Groeneveld’ showed a significantly reduced content of extractives compared to the non-infected trees, probably due to negatively impaired photosynthetic and metabolic processes.

## 5. Conclusions

Two Dutch elm hybrids, ‘Groeneveld’ and ‘Dodoens’, showing different vascular architecture in the secondary xylem and a contrasting tolerance to DED, were investigated in their vascular responses to the artificial inoculation with the hybrid isolate of *O*. *novo-ulmi* ssp. *americana* × *novo-ulmi*. The poor and delayed response of ‘Groeneveld’ trees to DED infection through changes to its vascular characteristics prevented neither the rapid fungal spread nor the massive colonisation of the secondary xylem tissues resulting in the death of the infected trees. ‘Dodoens’ trees responded to DED infection immediately in the earlywood through a severe reduction in the vessel lumen area and the vessel size : number ratio. A trend in the increasing density of vessels showing a reduced size was maintained not only in the latewood that was formed in the year of infection but also in the earlywood that was formed in the consecutive year.

## Figures and Tables

**Table 1:** Earlywood vascular anatomy in the examined Dutch elm hybrids.

Trait	‘Groeneveld’ Non-Infected (6th Annual Ring)	‘Groeneveld’ Infected (6th Annual Ring)	‘Dodoens’ Non-Infected (6th Annual Ring)	‘Dodoens’ Infected (6th Annual Ring)	‘Dodoens’ Non-Infected (7th Annual Ring)	‘Dodoens’ Infected (7th Annual Ring)
Vessel lumen area (10^−4^ mm^2^)	38.37 (1.50–237.22)	36.83 (1.64–235.92) (0.0001) *	53.54 (1.54–347.79)	12.70 (1.39–251.65) (0.0001) *	44.30 (1.35–322.65)	7.87 (1.35–202.72) (0.0001) *
Number of vessels (per mm^2^)	116.04 ± 16.79 cd	129.67 ± 24.12 c	64.75 ± 11.36 e	181.08 ± 32.90 b	89.55 ± 8.29 de	274.86 ± 46.91 a
*F* (mm^2^)	0.44 ± 0.03 a	0.37 ± 0.08 b	0.36 ± 0.04 b	0.23 ± 0.01 c	0.39 ± 0.02 ab	0.21 ± 0.03 c
*S* (10^−6^ mm^4^)	34.77 ± 11.31 bc	26.46 ± 20.15 cd	89.77 ± 26.55 a	7.55 ± 2.47 de	50.14 ± 10.41 b	2.94 ± 0.82 e
Vessel radial diameter (μm)	126.47 ± 37.32 b	120.10 ± 32.76 bc	153.41 ± 71.25 a	107.73 ± 50.57 c	161.78 ± 43.31 a	79.69 ± 40.97 d
Vessel tangential diameter (μm)	91.37 ± 28.43 c	90.12 ± 23.47 c	116.08 ± 49.20 b	80.49 ± 34.48 c	128.53 ± 30.46 a	65.81 ± 31.34 d

*F*, vessel lumen fraction; *S*, vessel size : number ratio. Data represent means ± sd. Mean values followed by the same letters, a–e within the same row across hybrids, are not significantly different at *P* < 0.05. * Data for vessel lumen area are presented as means with range values plus *P*-value for pairwise treatment comparisons.

**Table 2:** Bordered pit anatomy of earlywood vessels in the examined Dutch elm hybrids.

Trait	‘Groeneveld’ Non-Infected (6th Annual Ring)	‘Groeneveld’ Infected (6th Annual Ring)	‘Dodoens’ Non-Infected (6th Annual Ring)	‘Dodoens’ Infected (6th Annual Ring)	‘Dodoens’ Non-Infected (7th Annual Ring)	‘Dodoens’ Infected (7th Annual Ring)
Aperture area (μm^2^)	2.86 ± 0.94 c	2.91 ± 1.11 c	4.09 ± 0.95 a	3.25 ± 1.17 bc	3.46 ± 1.10 b	2.25 ± 1.17 d
Maximum diameter of aperture (μm)	3.09 ± 0.66 a	3.07 ± 0.79 a	3.02 ± 0.39 a	2.88 ± 0.66 a	2.84 ± 0.43 a	2.27 ± 0.55 b
Minimum diameter of aperture (μm)	1.32 ± 0.22 d	1.39 ± 0.23 d	1.90 ± 0.20 a	1.58 ± 0.25 c	1.71 ± 0.29 b	1.40 ± 0.36 d
Circularity ratio of aperture	0.44 ± 0.09 d	0.48 ± 0.13 c	0.63 ± 0.05 a	0.57 ± 0.11 b	0.60 ± 0.08 ab	0.62 ± 0.11 a
Abundance (per 1000 μm^2^ of wall)	11.24 ± 1.76 c	13.19 ± 1.60 ab	12.76 ± 1.99 ab	12.44 ± 1.36 bc	11.37 ± 1.27 c	13.77 ± 2.24 a

Data represent means ± sd. Mean values followed by the same letters, a–d within the same row across hybrids, are not significantly different at *P* < 0.05.

**Table 3:** Latewood vascular anatomy in the examined Dutch elm hybrids.

Trait	‘Groeneveld’ Non-Infected (6th Annual Ring)	‘Groeneveld’ Infected (6th Annual Ring)	‘Dodoens’ Non-Infected (6th Annual Ring)	‘Dodoens’ Infected (6th Annual Ring)	‘Dodoens’ Non-Infected (7th Annual Ring)	‘Dodoens’ Infected (7th Annual Ring)
Vessel lumen area (10^–4^ mm^2^)	7.08 (0.88–31.31)	5.43 (0.85–30.41) (0.0001) *	4.11 (0.86–27.93)	1.93 (0.85–42.06) (0.0001) *	3.09 (0.85–19.26)	2.41 (0.84–11.05) (0.0001) *
Number of vessels (per mm^2^)	196.76 ± 43.00 d	379.61 ± 67.88 bc	364.94 ± 45.00 bc	639.96 ± 116.59 a	424.72 ± 93.64 b	302.03 ± 40.41 c
*F* (mm^2^)	0.14 ± 0.01 b	0.19 ± 0.02 a	0.15 ± 0.02 ab	0.12 ± 0.01 b	0.14 ± 0.06 b	0.07 ± 0.01 c
*S* (10^–6^ mm^4^)	4.11 ± 2.03 a	1.51 ± 0.94 b	1.17 ± 0.35 b	0.32 ± 0.10 b	0.79 ± 0.11 b	0.82 ± 0.16 b
Vessel radial diameter (μm)	37.19 ± 12.29 a	38.21 ± 17.00 a	29.05 ± 10.19 b	17.50 ± 5.86 d	25.00 ± 8.04 c	18.16 ± 6.05 d
Vessel tangential diameter (μm)	31.02 ± 9.43 b	33.84 ± 13.56 a	25.98 ± 8.45 c	17.79 ± 5.95 f	22.60 ± 6.28 d	20.33 ± 5.69 e

*F*, vessel lumen fraction; *S*, vessel size : number ratio. Data represent means ± sd. Mean values followed by the same letters, a–f within the same row across hybrids, are not significantly different at *P* < 0.05. * Data for vessel lumen area are presented as means with range values plus *P*-value for pairwise treatment comparisons.

## Data Availability

Data are available on request from the corresponding author.

## Supplementary Materials

The following supporting information can be downloaded at: https://www.mdpi.com/article/10.3390/jof8030215/s1, Figure S1: The proportion of earlywood vessels in individual vessel lumen area classes determined for both non-infected and infected trees. (A) The sixth annual growth ring of ‘Groeneveld’ trees. (B) The sixth annual growth ring of ‘Dodoens’ trees. (C) The seventh annual growth ring of ‘Dodoens’ trees. Figure S2: Scanning electron microscopy image of inorganic crystal composed of calcium and silicon inside the earlywood vessel (upper image, tangential section, scale bar = 20 μm) accompanied by the representative energy-dispersive X-ray spectrum showing the elemental composition (bottom). Figure S3: Contrasting autofluorescence intensities for compounds deposited in the cells adjacent to the invaded vessels of earlywood. (A) The sixth annual growth ring of the infected ‘Groeneveld’ tree. (B) The sixth annual growth ring of the infected ‘Dodoens’ tree. Cross-sections, scale bars = 200 μm. Figure S4: The proportion of latewood vessels in individual vessel lumen area classes determined for both non-infected and infected trees. (A) The sixth annual growth ring of ‘Groeneveld’ trees. (B) The sixth annual growth ring of ‘Dodoens’ trees. (C) The seventh annual growth ring of ‘Dodoens’ trees. Video S1: X-ray micro-computed tomography radial imaging of fungal colonisation in earlywood tissues of the sixth annual growth ring of the infected ‘Groeneveld’ tree. Video S2: X-ray micro-computed tomography radial imaging of fungal colonisation in earlywood tissues of the sixth annual growth ring of the infected ‘Dodoens’ tree. Video S3: X-ray micro-computed tomography radial imaging of the seventh annual growth ring of the infected ‘Dodoens’ tree showing the restricted occurrence of fungal hyphae in earlywood tissues.
