# Supplementary material for: Different Responses in Vascular Traits between Dutch Elm Hybrids with a Contrasting Tolerance to Dutch Elm Disease
Source: J Fungi (Basel). 2022 Feb 22;8(3):215. doi: 10.3390/jof8030215 (PMC8954630; doi:10.3390/jof8030215)
Supplement: Supplementary file 1 [file jof-08-00215-s001.zip › Supplementary Figures.pdf]

Supplementary Figures

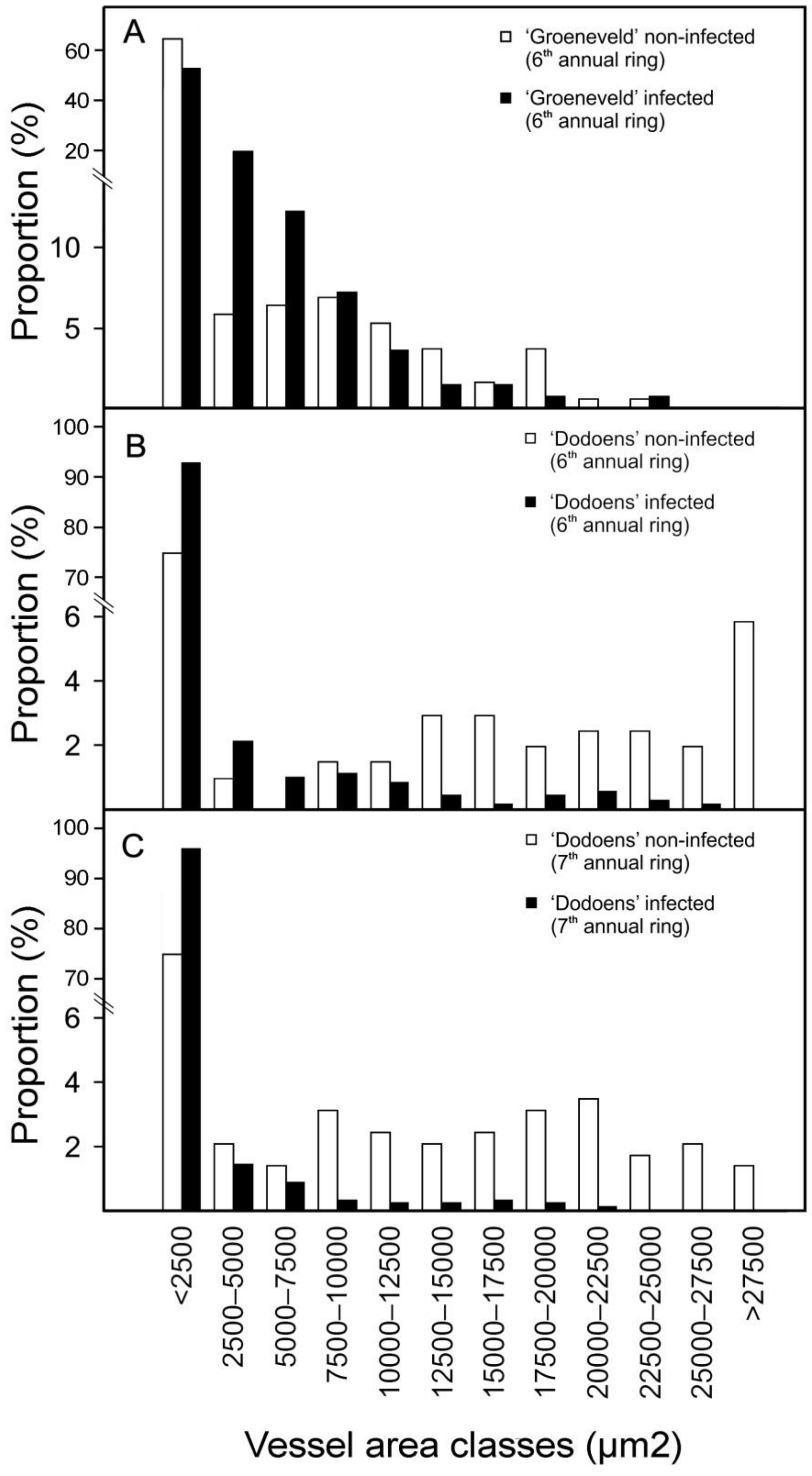

Figure S1. The proportion of earlywood vessels in individual vessel lumen area classes determined for both non-infected and infected trees. (**A**) The sixth annual growth ring of 'Groeneveld' trees. (**B**) The sixth annual growth ring of 'Dodoens' trees. (**C**) The seventh annual growth ring of 'Dodoens' trees.

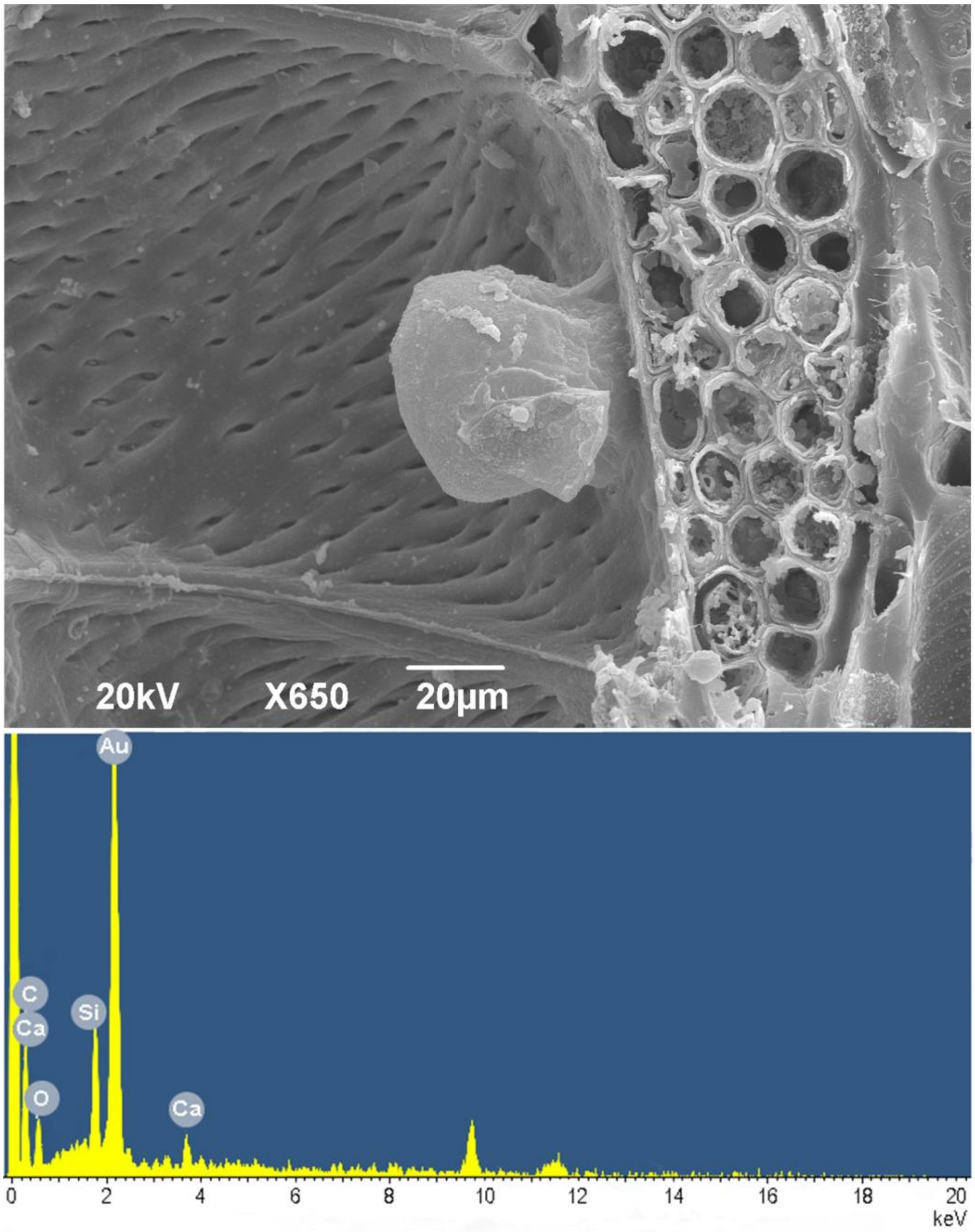

Figure S2. Scanning electron microscopy image of inorganic crystal composed of calcium and silicon inside the earlywood vessel (upper image, tangential section, scale bar = 20 μm) accompanied by the representative energy-dispersive X-ray spectrum showing the elemental composition (bottom).

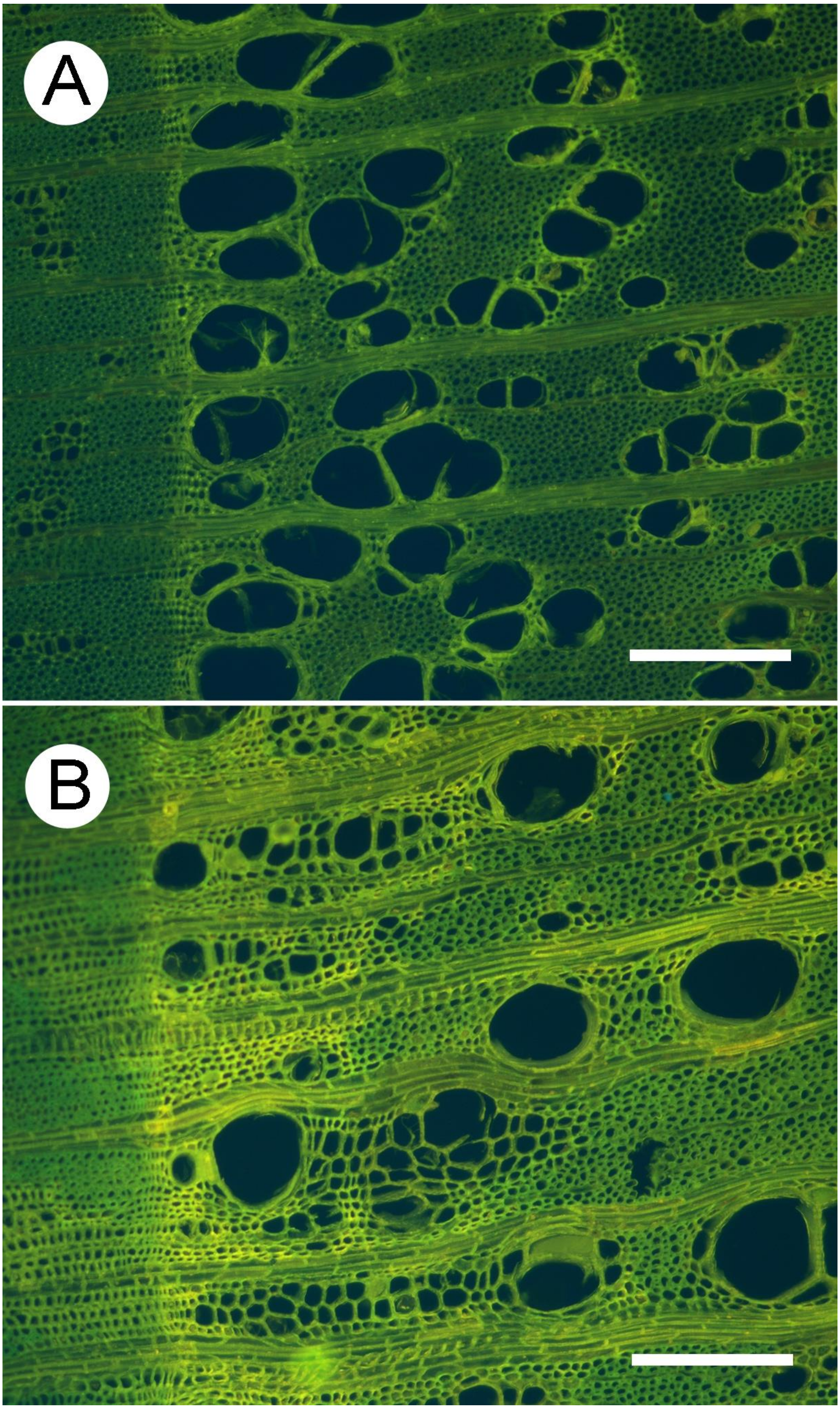

Figure S3. Contrasting autofluorescence intensities for compounds deposited in the cells adjacent to the invaded vessels of the earlywood. (**A**) The sixth annual growth ring of the infected 'Groeneveld' tree. (**B**) The sixth annual growth ring of the infected 'Dodoens' tree. Cross-sections, scale bars = 200 µm.

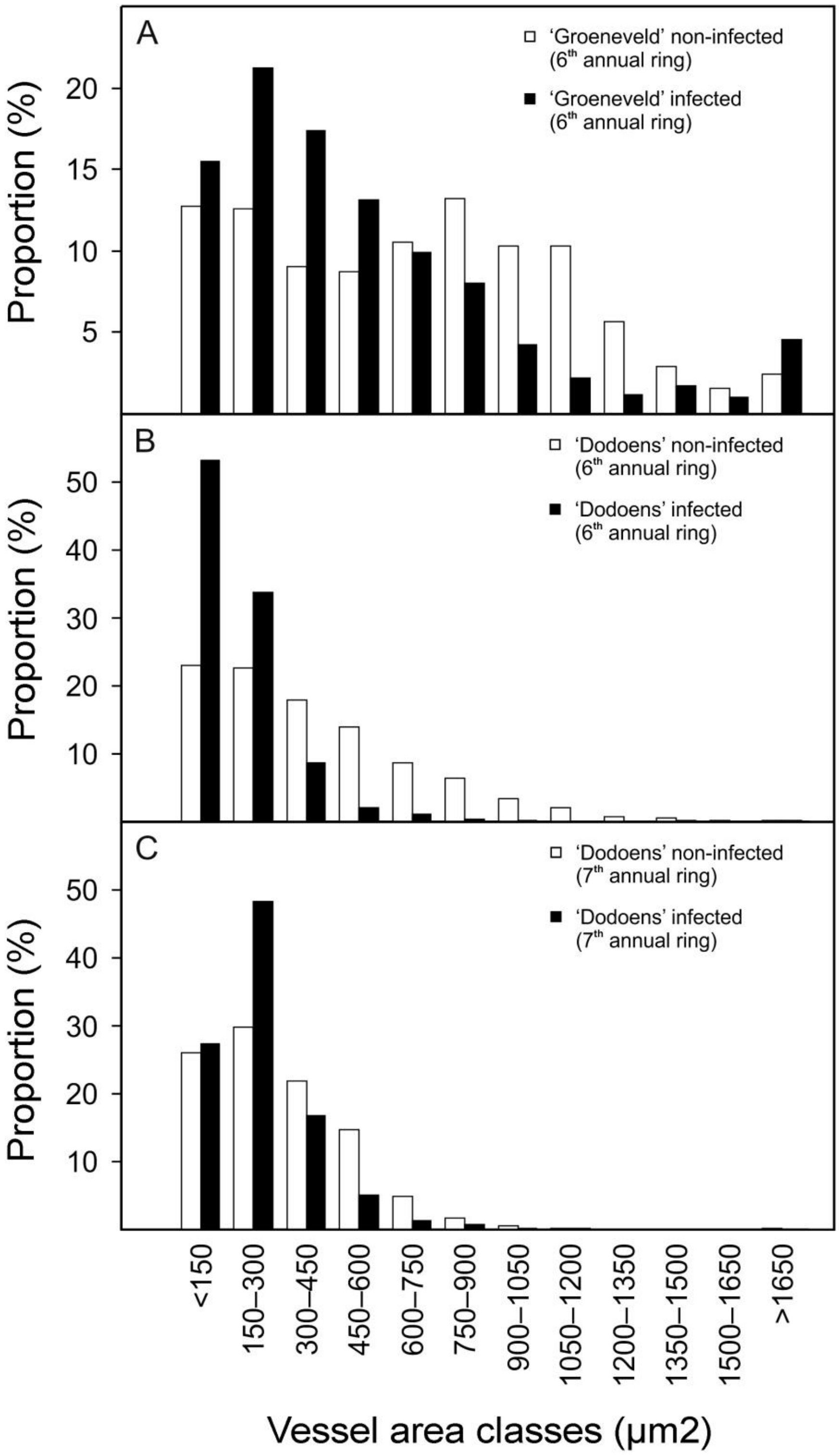

Figure S4. The proportion of latewood vessels in individual vessel lumen area classes determined for both non-infected and infected trees. (**A**) The sixth annual growth ring of 'Groeneveld' trees. (**B**) The sixth annual growth ring of 'Dodoens' trees. (**C**) The seventh annual growth ring of 'Dodoens' trees.
